# Predictors of training-related improvement in visuomotor performance in patients with multiple sclerosis: A behavioural and MRI study

**DOI:** 10.1177/1352458520943788

**Published:** 2020-08-04

**Authors:** Ilona Lipp, Catherine Foster, Rachael Stickland, Eleonora Sgarlata, Emma C Tallantyre, Alison E Davidson, Neil P Robertson, Derek K Jones, Richard G Wise, Valentina Tomassini

**Affiliations:** Division of Psychological Medicine and Clinical Neurosciences, School of Medicine, Cardiff University, Cardiff, UK/Cardiff University Brain Research Imaging Centre (CUBRIC), School of Psychology, Cardiff University, Cardiff, UK/Department of Neurophysics, Max Planck Institute for Human Cognitive and Brain Sciences, Leipzig, Germany; Cardiff University Brain Research Imaging Centre (CUBRIC), School of Psychology, Cardiff University, Cardiff, UK; Cardiff University Brain Research Imaging Centre (CUBRIC), School of Psychology, Cardiff University, Cardiff, UK; Division of Psychological Medicine and Clinical Neurosciences, School of Medicine, Cardiff University, Cardiff, UK/Cardiff University Brain Research Imaging Centre (CUBRIC), School of Psychology, Cardiff University, Cardiff, UK; Division of Psychological Medicine and Clinical Neurosciences, School of Medicine, Cardiff University, Cardiff, UK/Helen Durham Centre for Neuroinflammation, University Hospital of Wales, Cardiff, UK; Division of Psychological Medicine and Clinical Neurosciences, School of Medicine, Cardiff University, Cardiff, UK/Cardiff University Brain Research Imaging Centre (CUBRIC), School of Psychology, Cardiff University, Cardiff, UK; Division of Psychological Medicine and Clinical Neurosciences, School of Medicine, Cardiff University, Cardiff, UK/Helen Durham Centre for Neuroinflammation, University Hospital of Wales, Cardiff, UK; Cardiff University Brain Research Imaging Centre (CUBRIC), School of Psychology, Cardiff University, Cardiff, UK; Cardiff University Brain Research Imaging Centre (CUBRIC), School of Psychology, Cardiff University, Cardiff, UK/Institute for Advanced Biomedical Technologies (ITAB), Department of Neurosciences, Imaging and Clinical Sciences, University ‘G. d’Annunzio’ of Chieti-Pescara, Chieti, Italy; Division of Psychological Medicine and Clinical Neurosciences, School of Medicine, Cardiff University, Cardiff, UK/Cardiff University Brain Research Imaging Centre (CUBRIC), School of Psychology, Cardiff University, Cardiff, UK/Helen Durham Centre for Neuroinflammation, University Hospital of Wales, Cardiff, UK/Institute for Advanced Biomedical Technologies (ITAB), Department of Neurosciences, Imaging and Clinical Sciences, University ‘G. d’Annunzio’ of Chieti-Pescara, Chieti, Italy

**Keywords:** Multiple sclerosis, brain plasticity, predictors, functional recovery, MRI, cognition, disease-modifying treatment

## Abstract

**Background:**

The development of tailored recovery-oriented strategies in multiple sclerosis requires early identification of an individual’s potential for functional recovery.

**Objective:**

To identify predictors of visuomotor performance improvements, a proxy of functional recovery, using a predictive statistical model that combines demographic, clinical and magnetic resonance imaging (MRI) data.

**Methods:**

Right-handed multiple sclerosis patients underwent baseline disability assessment and MRI of the brain structure, function and vascular health. They subsequently undertook 4 weeks of right upper limb visuomotor practice. Changes in performance with practice were our outcome measure. We identified predictors of improvement in a *training set* of patients using lasso regression; we calculated the best performing model in a *validation set* and applied this model to a *test set.*

**Results:**

Patients improved their visuomotor performance with practice. Younger age, better visuomotor abilities, less severe disease burden and concurrent use of preventive treatments predicted improvements. Neuroimaging localised outcome-relevant sensory motor regions, the microstructure and activity of which correlated with performance improvements.

**Conclusion:**

Initial characteristics, including age, disease duration, visuo-spatial abilities, hand dexterity, self-evaluated disease impact and the presence of disease-modifying treatments, can predict functional recovery in individual patients, potentially improving their clinical management and stratification in clinical trials. MRI is a correlate of outcome, potentially supporting individual prognosis.

## Introduction

Prediction of an individual patient’s capacity for functional improvements is relevant for patient management in multiple sclerosis (MS), as it allows personalised recovery interventions, rationalisation of health resource allocation and improved stratification of patients in clinical trials.^1^ Currently, the prediction of functional recovery in the individual case is based solely on clinical experience and thus remains largely variable and inaccurate.^2^

The individual’s potential for functional recovery results from the complex interaction of age, MS damage and disability, residual abilities and pre-morbid reserve, all of which can be captured by clinical measures and by magnetic resonance imaging (MRI) features.^3^ Indeed, characteristics such as age and disease severity can play a role in disability development and worsening,^4,5^ as well as in determining individual outcomes during recovery-oriented interventions.^6–8^ Brain damage and reserve, as assessed by MRI, can also influence the individual’s functional potential.^3^

In this study, we combined demographic with baseline clinical and MRI measures to identify predictors of functional recovery in patients with MS. We used upper limb performance improvements on a visuomotor task as a proxy for functional recovery.^3,9^ *Firstly*, in a cohort of MS patients, we employed exploratory statistical modelling by combining measures of initial disability with functional and structural neuroimaging metrics, acquired before 4 weeks of home practice of a standardised upper limb visuomotor task.^10^ *Secondly*, using this predictive model of functional recovery, we identified predictors of individual performance improvements. *Thirdly*, we validated our predictive model and results in an independent patient cohort.

## Methods

### Patients and study design

We recruited right-handed MS patients^11^ aged 18–60 years, with retained use of their right upper limb, no relapse or change in treatment for at least 3 months before study entry, and no other neurological or psychiatric conditions. Patients were recruited within the Helen Durham Centre for Neuroinflammation, University Hospital of Wales, Cardiff, UK. The study was approved by the NHS South-West Ethics Committee. All patients provided written informed consent.

At baseline, we collected demographic and clinical measures (Table 1), as well as functional and structural MRI (Table 2). Patients were instructed to practise a visuomotor task at home and subsequently returned to be clinically assessed.

**Table 1.** Demographic and clinical characteristics for the whole cohort (*N* = 118), as well as for the three randomly assigned groups.

Characteristics	Whole cohort (*N* = 118)	Training set (*n* = 71)	Validation set (*n* = 24)	Test set (*n* = 23)	*p*
Age	44.55 ± 9.47	44.85 ± 9.93	45.00 ± 8.78	43.17 ± 8.95	0.74
Gender (F/M)	74/44	44/27	16/8	14/9	0.90
Education (years)	16.14 ± 4.27	15.69 ± 3.90	17.50 ± 5.45	16.09 ± 3.87	0.20
EDSS score	4.0 ± 1.4	4.0 ± 1.5	4.0 ± 2.0	4.0 ± 2.0	0.59
Disease duration (years)	12.58 ± 7.57	13.31 ± 8.02	11.67 ± 6.82	11.30 ± 6.89	0.44
Clinical course (RRMS/PMS)	93/25	57/14	19/5	17/6	0.81
DMT (present/absent)	38/80	20/51	6/18	12/11	0.07
R hand grip strength (kg)	29.28 ± 11.91	29.75 ± 12.80	28.29 ± 9.94	28.87 ± 11.29	0.86
L hand grip strength (kg)	28.83 ± 12.31	28.77 ± 13.53	28.04 ± 8.93	29.85 ± 11.72	0.88
R 9-HPT (median/IQR)	22.40 ± 6.75	22.80 ± 7.57	23.20 ± 3.12	21.65 ± 6.90	0.79
L 9-HPT (median/IQR)	23.25 ± 5.31	23.25 ± 5.75	23.57 ± 4.10	22.75 ± 4.11	0.79
T25-FW (median/IQR)	5.65 ± 2.71	5.62 ± 2.70	5.40 ± 1.89	5.83 ± 3.30	0.61
SRT-C	32.32 ± 18.52	34.30 ± 21.09	28.39 ± 13.52	30.13 ± 13.30	0.34
SRT-L	43.59 ± 14.83	44.39 ± 15.67	42.74 ± 11.95	41.96 ± 15.18	0.76
Delayed SRT	8.39 ± 2.55	8.52 ± 2.48	8.00 ± 2.02	8.39 ± 3.26	0.69
SPART	6.07 ± 1.69	6.00 ± 1.69	5.93 ± 1.59	6.46 ± 1.80	0.47
Delayed SPART	6.33 ± 2.36	6.24 ± 2.35	6.21 ± 2.28	6.74 ± 2.54	0.66
SDMT	52.25 ± 10.48	52.92 ± 10.72	50.58 ± 8.46	51.96 ± 11.79	0.64
PASAT 3s	40.47 ± 13.93	42.21 ± 12.71	39.75 ± 11.57	35.83 ± 18.56	0.16
WLG	25.86 ± 7.45	25.66 ± 6.71	27.33 ± 7.74	24.96 ± 9.24	0.52
MFIS	39.35 ± 21.16	41.04 ± 22.56	35.10 ± 16.37	38.57 ± 21.28	0.49
STAI	40.77 ± 11.57	41.33 ± 12.48	38.36 ± 9.82	41.56 ± 10.35	0.52
BDI	12.39 ± 10.45	13.26 ± 10.88	8.47 ± 6.39	13.82 ± 11.88	0.12
MSIS-29	65.15 ± 29.75	66.21 ± 31.43	58.73 ± 19.65	68.58 ± 33.16	0.47
Baseline accuracy (%correct responses)	29.59 ± 23.59	30.31 ± 25.39	25.26 ± 20.88	31.88 ± 20.63	0.58
Baseline RT (ms)	382.86 ± 56.00	383.39 ± 57.49	379.85 ± 49.61	384.37 ± 59.82	0.96

EDSS: Extended Disability Status Scale; RRMS: relapsing-remitting multiple sclerosis; PMS: progressive multiple sclerosis; DMT: disease-modifying treatment; R: right; L: left; 9-HPT: nine-hole peg test; IQR: interquartile range; T25-FW: timed 25-foot walk; SRT-C: Selective Reminding Test Consistent Retrieval; SRT-L: Selective Reminding Test Long-Term Storage; SPART: Spatial Recall Test; SDMT: Symbol Digit Modalities Test; PASAT: Paced Auditory Serial Addition Test; WLG: Word List Generation; MFIS: Modified Fatigue Impact Scale; STAI: State Trait Anxiety Inventory; BDI: Beck Depression Inventory; MSIS-29: 29-item Multiple Sclerosis Impact Scale; RT: reaction time; ANOVA: analysis of variance.

Unless otherwise indicated, the provided descriptive statistics are means and standard deviations. For comparison between the three groups, chi-square tests were performed for categorical variables, Kruskal–Wallis tests for skewed variables (9-HPT, T25-FW) and one-way ANOVAs for the rest. *p* values for group differences are provided.

**Table 2.** MRI characteristics for the whole cohort (*N* = 118), as well as for the three randomly assigned groups. Measures of cortical thickness and percent (%) damaged voxels were not normalised to the intracranial volume.

	All (*N* = 118)	Training set (*n* = 71)	Validation set (*n* = 24)	Test set (*n* = 23)	*p*
Structural measures: volume fractions (normalised to intracranial volume)					
Whole brain volume	69.77 ± 2.94	69.99 ± 3.07	69.22 ± 2.98	69.66 ± 2.52	0.54
Cortical GM	27.76 ± 2.08	27.88 ± 2.25	27.35 ± 1.93	27.83 ± 1.66	0.56
L average cortical thickness (mm)	2.48 ± 0.14	2.48 ± 0.17	2.45 ± 0.09	2.50 ± 0.09	0.56
R average cortical thickness (mm)	2.49 ± 0.14	2.50 ± 0.17	2.47 ± 0.11	2.50 ± 0.09	0.66
L cerebellar cortex	3.39 ± 0.31	3.37 ± 0.29	3.41 ± 0.27	3.41 ± 0.42	0.81
L thalamus	0.46 ± 0.06	0.46 ± 0.06	0.45 ± 0.06	0.47 ± 0.06	0.29
L caudate	0.20 ± 0.02	0.20 ± 0.02	0.20 ± 0.02	0.20 ± 0.02	0.51
L putamen	0.28 ± 0.04	0.28 ± 0.04	0.27 ± 0.05	0.28 ± 0.04	0.85
L pallidum	0.07 ± 0.01	0.07 ± 0.01	0.07 ± 0.01	0.07 ± 0.01	0.95
Brainstem	1.25 ± 0.12	1.25 ± 0.11	1.22 ± 0.13	1.28 ± 0.11	0.24
R cerebellar cortex	3.50 ± 0.33	3.48 ± 0.32	3.51 ± 0.33	3.53 ± 0.36	0.78
R thalamus	0.40 ± 0.05	0.40 ± 0.05	0.38 ± 0.05	0.39 ± 0.05	0.14
R caudate	0.19 ± 0.03	0.19 ± 0.03	0.19 ± 0.02	0.18 ± 0.03	0.23
R putamen	0.26 ± 0.04	0.26 ± 0.03	0.25 ± 0.04	0.27 ± 0.04	0.28
R pallidum	0.07 ± 0.01	0.08 ± 0.01	0.07 ± 0.01	0.08 ± 0.01	0.64
GM microstructural damage (% damaged voxels)	0.09 ± 0.09	0.09 ± 0.08	0.10 ± 0.10	0.10 ± 0.09	0.74
Structural measures: WM measures					
Lesion volume (cm^3^, median/IQR)	2.84 ± 3.86	2.81 ± 4.03	2.59 ± 5.76	2.87 ± 2.84	0.84
FA in NAWM (*z* score)	−0.29 ± 0.30	−0.28 ± 0.29	−0.30 ± 0.27	−0.30 ± 0.38	0.92
FA in T2L (*z* score)	−1.20 ± 0.48	−1.25 ± 0.48	−1.17 ± 0.50	−1.10 ± 0.49	0.44
RD in NAWM (*z* score)	0.49 ± 0.53	0.45 ± 0.52	0.59 ± 0.43	0.51 ± 0.68	0.57
RD in T2L (*z* score)	2.68 ± 1.28	2.75 ± 1.35	2.73 ± 1.19	2.41 ± 1.17	0.53
MTR in NAWM (*z* score)	−0.23 ± 0.82	−0.18 ± 0.69	−0.26 ± 0.94	−0.35 ± 1.05	0.66
MTR in T2L (*z* score)	−2.01 ± 1.32	−2.00 ± 1.25	−2.08 ± 1.53	−1.98 ± 1.36	0.96
Cerebral blood flow					
L cerebellar cortex	39.60 ± 13.62	38.75 ± 11.95	37.12 ± 12.16	45.18 ± 18.62	0.11
R cerebellar cortex	37.65 ± 12.50	36.93 ± 11.72	35.45 ± 11.69	42.51 ± 15.05	0.14
L caudate	23.78 ± 8.46	23.55 ± 8.54	21.28 ± 6.95	27.42 ± 8.92	0.05
R caudate	30.08 ± 9.23	30.65 ± 9.41	26.69 ± 8.02	32.14 ± 9.37	0.11
L putamen (median/IQR)	48.15 ± 13.02	48.27 ± 13.80	45.32 ± 12.05	50.83 ± 17.34	0.26
R putamen (median/IQR)	42.77 ± 13.01	43.20 ± 12.95	40.94 ± 11.63	45.04 ± 14.98	0.17
L thalamus	38.29 ± 12.51	37.24 ± 10.29	34.92 ± 11.74	45.56 ± 16.98	0.01
R thalamus	35.16 ± 10.79	34.56 ± 9.18	32.65 ± 12.81	39.95 ± 12.13	0.07
L pallidum (median/IQR)	39.82 ± 14.26	40.08 ± 14.55	38.71 ± 14.67	38.73 ± 20.92	0.77
R pallidum (median/IQR)	40.58 ± 10.58	41.21 ± 9.54	35.69 ± 9.39	43.46 ± 13.89	0.05
L cerebral cortex	47.52 ± 13.85	46.32 ± 13.07	45.40 ± 12.69	53.78 ± 16.28	0.08
R cerebral cortex	44.87 ± 12.73	44.16 ± 11.70	42.02 ± 11.74	50.42 ± 15.71	0.08
Functional measures: BOLD (cope) SRT Task vs Rest					
L lateral pre/postcentral gyrus	277 ± 151	279 ± 132	269 ± 183	279 ± 176	0.96
L medial precentral gyrus	130 ± 80	133 ± 65	128 ± 85	125 ± 114	0.90
R lateral precentral gyrus	219 ± 111	216 ± 107	220 ± 90	226 ± 145	0.93
R cerebellar lobe VI	270 ± 118	269 ± 115	274 ± 110	268 ± 139	0.98
R cerebellar lobe VIII	275 ± 119	290 ± 110	269 ± 115	232 ± 141	0.13
R V5	298 ± 115	304 ± 110	293 ± 99	281 ± 148	0.69
Functional measures: BOLD (cope) Sequence-specific signal changes					
Precuneus	3.86 ± 16.04	6.81 ± 15.27	−5.62 ± 16.47	4.68 ± 14.64	<0.001
R IPS	−8.62 ± 21.99	−6.30 ± 19.36	−19.08 ± 31.79	−4.70 ± 12.43	0.03
R V1	−5.56 ± 21.25	−4.65 ± 21.19	−12.96 ± 24.06	−0.45 ± 16.49	0.12
Outcome-relevant measures					
FA	0.47 ± 0.03	0.47 ± 0.04	0.46 ± 0.03	0.47 ± 0.04	0.85
BOLD	128.16 ± 99.95	121.83 ± 92.72	123.49 ± 85.26	153.68 ± 133.14	0.42

MRI: magnetic resonance imaging; GM: grey matter; L: left; R: right; WM: white matter; IQR: interquartile range; FA: fractional anisotropy; NAWM: normal-appearing white matter; T2L: T2-hyperintense white matter lesions; RD: radial diffusivity; MTR: magnetisation transfer ratio; BOLD: blood oxygenation level dependent; cope: contrast parameter estimates (BOLD activation is presented in arbitrary units); SRT: serial reaction time; V1: area V1; V5: area V5; IPS: intraparietal sulcus.

Unless otherwise indicated, descriptive statistics provided are means and standard deviations. For comparison between the three groups, chi-square tests were performed for categorical variables, Kruskal–Wallis tests for skewed variables (i.e. lesion volume, CBF in the putamen and pallidum), and one-way ANOVA for the rest. *p* values for group differences are provided. *GM microstructural damage* represents the proportion of GM with damaged voxels, whereby damage was assessed through a voxel-wise comparison of the MTR to healthy control tissue. FA, RD and MTR measures are reported as *z* scores, derived from a voxel-wise comparison to healthy control tissue. Reductions or increases in sequence-specific activation changes are indicated by the sign of the measure. Outcome-relevant measures are functional measures derived from brain regions where correlations with outcome measures were found.

### Visuomotor task and home practice

**Figure 1.**
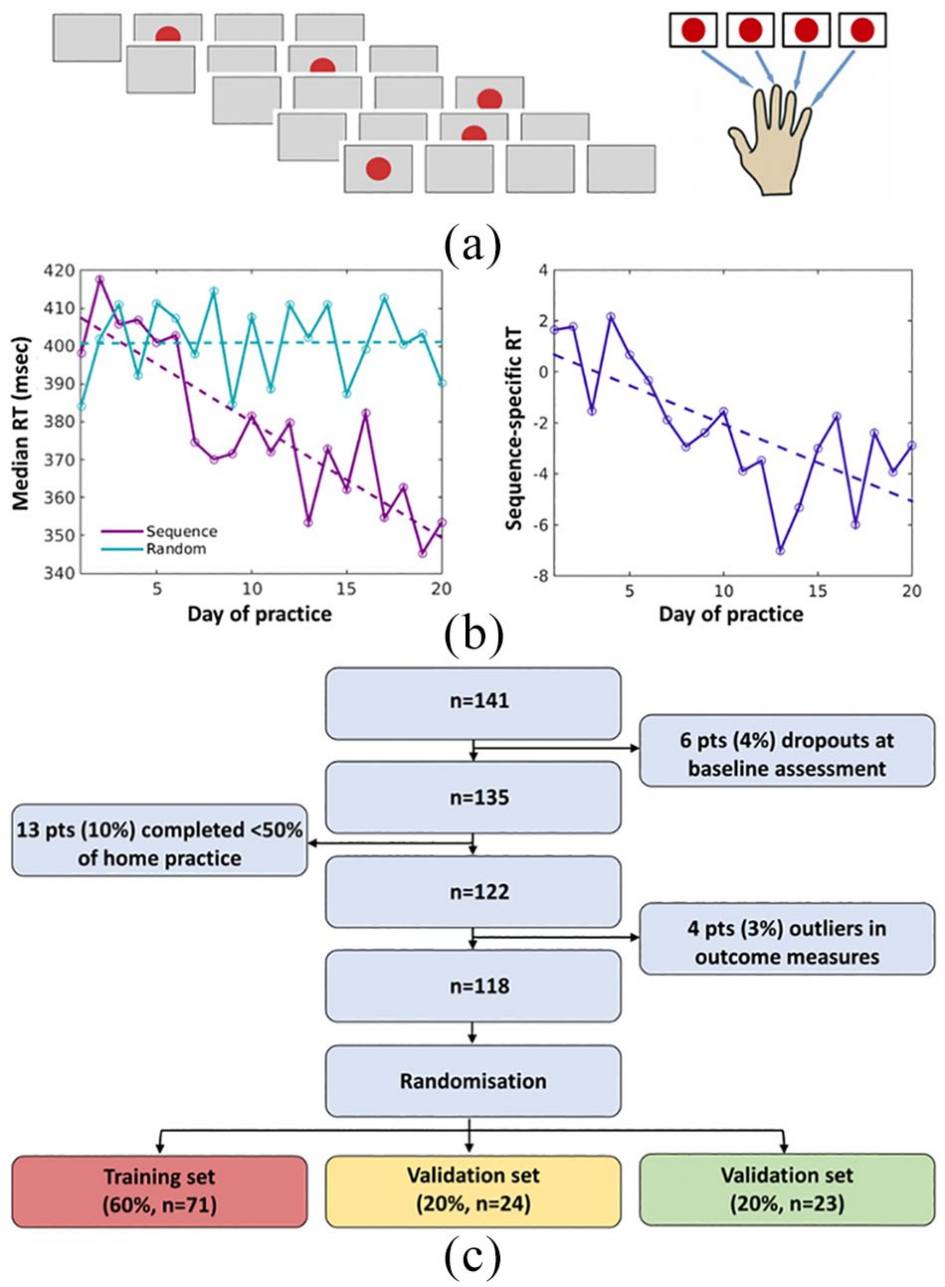
Experimental task and participants. (a) The serial reaction time (SRT) task stimuli consisted of a red circle that could appear in one of four positions horizontally aligned on the computer screen. Participants had to react to the location of the circle by pressing a button with the corresponding finger as quickly as possible. (b) Example of one patient’s performance, measured as the reaction time (RT), in Sequence and Random conditions over 20 days of home practice (*left*). A contrast measure between Sequence and Random blocks was computed to capture the Sequence-specific aspect of performance improvements. The RT change over the training period was linearly modelled using the slope of this change as the home-practice outcome measure for that individual patient (*right*). (c) Out of 141 recruited patients, 118 successfully completed the study and entered the analysis. For the predictive modelling, patients were randomly assigned to one of three groups: training set, validation set and test set. pts: patients.

We used a serial reaction time (SRT) task to probe recovery experimentally at baseline, while undergoing a brain MRI scan, and subsequently at home, on a laptop, daily for 5 days/week, for 4 weeks, with each practice session lasting for about 15 minutes (see Figure 1(a) and Supplementary Material for details on the SRT task design and presentation). For the home practice, patients were asked to complete a practice log sheet on paper and weekly phone calls were made by the study team to ensure compliance. During the home practice, the SRT task was presented on a laptop, on which the patient’s responses were recorded to be used for the analysis of performance improvements.

During the SRT task, stimuli were presented in the ‘Sequence’, ‘Random’ or ‘Rest’ condition. For each block of the SRT task, the number (accuracy) and median latency (reaction time (RT)) of correct responses were calculated. For each day of practice, the accuracy and RT across blocks were calculated (Figure 1(b), left). As accuracy can rapidly reach a plateau, we used RT to describe changing performance with home practice. Only patients who completed at least 50% of the scheduled sessions (10 days) were included in the analysis.

For each participant and day of practice, we compared RT in Sequence *versus* Random blocks using an unpaired *t* test in order to generate a contrast measure that represented Sequence-specific performance changes. For each participant, a linear model was fitted using *robustfit* of MATLAB, with practice day as the independent variable and the Sequence-specific contrast measure as the dependent variable. We used the individual slope of practice-related Sequence-specific RT changes as our outcome measure of visuomotor performance changes with home practice (Figure 1(b), right).

### Demographic and clinical characteristics

Demographic and clinical characteristics are indicated in Table 1. Disease duration was defined as the time (in years) between the onset of the first symptoms and the time of the study assessment. The date of disease onset was established from the patient’s clinical notes (whenever available) and confirmed during the study interview. Expanded Disability Status Scale (EDSS)^12^ and 29-item Multiple Sclerosis Impact Scale (MSIS-29)^13^ assessed disability and disease impact; Beck Depression Inventory (BDI),^14^ State Trait Anxiety Inventory (STAI)^15^ scale and Modified Fatigue Impact Scale (MFIS)^16^ quantified mood, anxiety and fatigue, respectively; nine-hole peg test (9-HPT)^17^ and timed 25-foot walk (T25-FW)^17^ characterised the limb function; Rao’s Brief Repeatable Battery^18^ probed cognition.

### MRI acquisition and analysis

**Figure 2.**
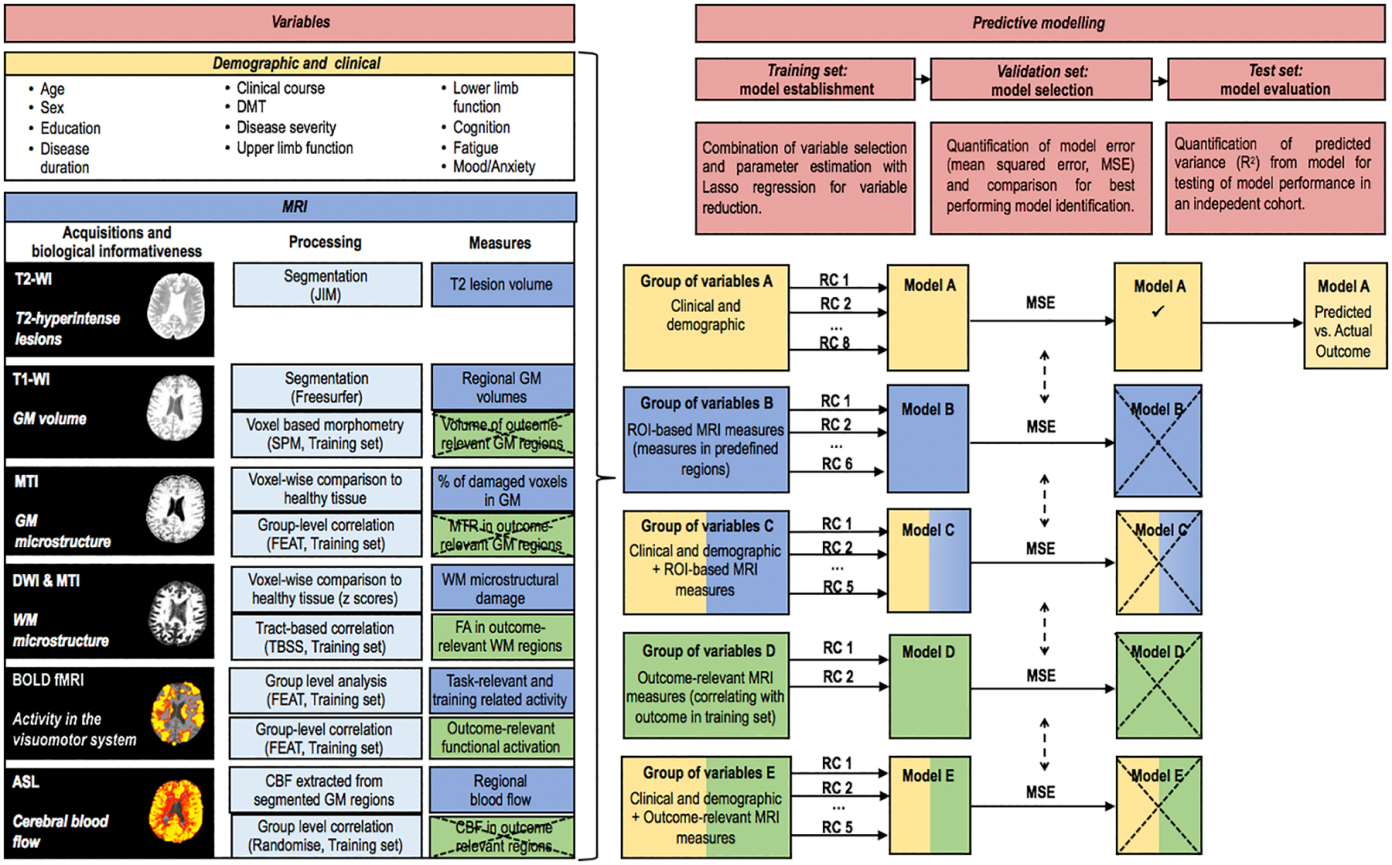
Overview of the analysis. All the demographic, clinical and MRI variables considered for the modelling are listed (*left*). For each type of the MRI variable, the acquisition sequence, the biological informativeness, the main processing steps and the extracted measures are indicated. ROI-based and outcome-relevant measures are indicated in blue and green boxes, respectively. Crossed boxes indicate non-significant results. The extracted measures that survived the statistical analysis were considered for the development of the predictive models. All variables were clustered in groups on the basis of their type (demographic and clinical, ROI-based MRI, outcome-relevant MRI) or combinations of types (*right*). First, within each group of variables (A–E), a lasso regression was performed in the *training set*, resulting in a number of retained variables and their corresponding regression coefficients. Second, these were applied to the *validation set* in order to quantify the modelling error (MSE). Third, the model with the lowest model error in the validation set (model A) was applied to the *test set* to assess the relationship between the outcome predicted by the model built in the training set and the actual outcome as measured in the test set. Crossed boxes indicate that the model did not survive the comparison of MSE. T2-WI: T2-weighted image; T1-WI: T1-weighted image; GM: grey matter; MTI: magnetisation transfer imaging; DWI: diffusion-weighted imaging; BOLD: blood oxygenation level dependent; ASL: arterial spin labelling; DMT: disease-modifying treatment; MSE: mean squared error; WM: white matter; SRT: serial reaction time; RC: regression coefficient; ROI: region of interest.

We acquired brain MRI scans on a 3-T MRI system (GE Medical Systems, Milwaukee, WI) using an eight-channel receive-only head radiofrequency (RF) coil. Detailed descriptions of the MRI protocols and analysis pipelines are shown in Figure 2 and Supplementary Material.

MRI measures that entered the predictive modelling were selected to capture as many aspects of MS damage as possible in a single scan session. We considered lesional and non-lesional damage, functional responses and cerebral blood flow (CBF). For all MRI modalities, we extracted measures from predefined regions of interest (ROIs; ROI-based MRI measures); functional magnetic resonance imaging (fMRI) measures were defined in task-relevant ROIs. For all MRI modalities, we also extracted measures from areas of significant correlations with home-practice outcome (outcome-relevant MRI measures; Table 2) for use in the models of prediction.

### Predictive modelling

To predict performance improvements from baseline data, we employed a statistical learning approach that included a random assignment of the patients to three groups: a training set (60%) to establish statistical models, a validation set (20%) to select the best performing model in independent data and a test set (20%) to evaluate the performance of the selected model. The three statistical sets underwent the same study procedures, including home practice.

Five groups of variables were established in the training set: *group A* that included demographic and clinical variables; *group B* that included ROI-based MRI measures; *group C* that included demographic and clinical, as well as ROI-based MRI measures; *group D* that included outcome-relevant MRI measures; and *group E* that included demographic and clinical, as well as outcome-relevant MRI measures.

### Data preparation

Prior to modelling, all variables were *z*-standardised using the mean and standard deviation (SD) from the training set. Missing values were mean-imputed. Patients with a home-practice outcome 1.5 interquartile ranges below the first quartile or above the third quartile of the distribution were excluded from modelling. Patients unable to complete T25-FW were assigned the value of the slowest patient who was able to complete the test. Variables with an absolute skewness > 2 were log transformed: the scores of 9-HPT and T25-FW, the measures of lesion volume and the CBF in the putamen and globus pallidus.

### Statistical modelling

Lasso regression was used for the linear modelling in the training set (MATLAB (v R2015a) function: *lasso*). This method performs variable selection and parameter estimation simultaneously.^19^ Lasso regression overcomes the overfitting problems that are associated with traditional multiple linear regression analyses, by employing a regularisation of hyperparameter lambda that penalises for the number of non-zero regression coefficients. Here, the optimal lambda was estimated by seven-fold cross-validation (repeated and averaged over five times) within the training set, finding lambda that minimises the mean squared error (MSE) in the left-out folds. Correlations between predicted and actual outcomes were calculated using Pearson correlation coefficients.

Lasso regression was applied to each of the five groups of variables in the training set for linear modelling (Figure 2), generating five linear regression models (models A–E), which consisted of a number of retained variables along with their parameter estimates (regression coefficients). To obtain an index of the predicted home-practice outcome for each patient, we applied the regression coefficients of each of the five models to the baseline demographic, clinical and MRI data. Selection of the best performing regression model occurred by comparing the MSE across models in the validation set. The MSE quantifies how well the predicted home-practice outcomes matched the measured home-practice outcomes, with a low MSE indicating a small difference between predicted and measured outcomes. Therefore, the model with the lowest MSE was deemed the model best performing in the validation set and was tested for predictive performance in the test set by quantifying the amount of variance (*R*^2^) in the actual home-practice outcome that could be explained by the predicted home-practice outcome.

### Data availability statement

Anonymised data may be shared with qualified investigators.

## Results

### Baseline characteristics

Out of 141 recruited MS patients, 19 did not complete the study, including 6 who did not comply with baseline study procedures (e.g. claustrophobia in the scanner) and 13 patients who did not complete the home training programme (Figure 1(c)). Out of 122 patients who completed the study, four patients were identified as outliers in the home-practice outcome and thus not considered for the modelling purposes (Figure 1(c)). Figure S1 shows the distributions of the demographic and clinical characteristics for the whole cohort of patients. Table 1 summarises the characteristics of the whole cohort (*N* = 118), as well as of the three groups of patients. There was no difference among the training, validation and test sets in their baseline demographic and clinical characteristics. Patients were on average in their mid-40s, mainly women and mildly to moderately disabled,^20^ as suggested by measures of global disability, limb function, cognition, anxiety, mood and fatigue. They had a wide range of disease duration, with many patients being untreated, but (as requested by the eligibility criteria) in a stable phase of their disease course.

### Changes in behavioural measures with home practice

Patients completed 18.8 ± 2.2 days of home practice (min = 11, max = 24). A two-way analysis of variance (ANOVA) showed a significant interaction between time and condition, indicating stronger visuomotor performance improvements in the Sequence than in the Random for both RT (*F*(1, 117) = 115, *p* < 0.001) and accuracy (*F*(1, 117) = 82, *p* < 0.001). There were significant Sequence-specific RT improvements with practice in the whole cohort of patients (mean ± SD = –0.45 ± 0.33; *t*(117) = –15, *p* < 0.0001). The three groups did not differ in the mean ± SD Sequence-specific RT changes (training set: –0.41 ± 0.32; validation set: –0.52 ± 0.37; test set: –0.49 ± 0.30; *p* = 0.29).

With practice, patients showed better performance in the 9-HPT and cognitive tests, as well as improved levels of mood and fatigue (Table 3). This improvement did not correlate with the extent of home training–related improvements (Table 3).

**Table 3.** Clinical measures and their changes over time.

Clinical variable	Session 1	Session 2	Session 2 vs session 1	*t*	*p*	*r*	*p*
R 9-HPT	25.4 ± 11.7	23.7 ± 9.9	−1.5 ± 4.3	−3.8	<0.001	−0.14	0.13
R hand grip strength	29.3 ± 11.9	29.5 ± 12.4	0.2 ± 4.5	0.6	0.56	0.097	0.30
L 9-HPT	26.1 ± 14.3	24.4 ± 7.1	−1.6 ± 8.6	−2.0	0.052	−0.18	0.05
L hand grip strength	28.8 ± 12.3	29.0 ± 11.8	0.3 ± 3.9	0.7	0.47	0.045	0.63
T25-FW	8.5 ± 10.2	7.9 ± 9.7	−0.04 ± 1.75	−0.21	0.83	0.14	0.13
SRT-C	32.3 ± 18.5	36.9 ± 15.7	4.8 ± 15.5	3.3	0.001	−0.10	0.29
SRT-L	43.6 ± 14.8	46.4 ± 13.2	3.1 ± 10.5	3.1	0.002	−0.067	0.47
Delayed SRT	8.4 ± 2.6	8.5 ± 2.8	0.2 ± 2.1	0.9	0.37	−0.089	0.34
SPART	6.1 ± 1.7	6.9 ± 1.8	0.8 ± 1.8	5.1	<0.001	0.008	0.93
Delayed SPART	6.3 ± 2.4	7.3 ± 2.3	1.0 ± 2.4	4.4	<0.001	0.038	0.68
SDMT	52.3 ± 10.5	55.5 ± 11.4	3.2 ± 6.0	5.6	<0.001	−0.064	0.50
WLG	25.9 ± 7.4	28.8 ± 8.7	2.6 ± 8.3	3.4	0.001	−0.043	0.65
PASAT 3s	40.5 ± 13.9	45.1 ± 12.8	4.6 ± 7.2	6.8	<0.001	−0.13	0.16
MSIS-29	65.2 ± 29.8	62.0 ± 27.9	−2.5 ± 11.1	−2.4	0.018	−0.086	0.36
MFIS	39.4 ± 21.2	35.0 ± 20.9	−3.9 ± 9.8	−4.3	<0.001	0.057	0.54
STAI	40.8 ± 11.6	39.1 ± 12.1	−1.5 ± 5.7	−2.8	0.006	0.16	0.10
BDI	12.4 ± 10.5	10.4 ± 10.3	−1.8 ± 4.6	−4.3	<0.001	0.045	0.63

R: right; 9-HPT: nine-hole peg test; L: left; T25-FW: timed 25-foot walk; SRT-C: Selective Reminding Test Consistent Retrieval; SRT-L: Selective Reminding Test Long-term Storage; SPART: Spatial Recall Test; SDMT: Symbol Digit Modalities Test; WLG: Word List Generation; PASAT: Paced Auditory Serial Addition Test; MSIS-29: 29-item Multiple Sclerosis Impact Scale; MFIS: Modified Fatigue Impact Scale; STAI: State Trait Anxiety Inventory; BDI: Beck Depression Inventory.

For each clinical variable, the group mean ± standard deviation values across 118 patients are provided separately for sessions 1 and 2, as well as for the difference between the two sessions. In order to test whether, across the whole group, there was a significant longitudinal change in behavioural measures, we performed a paired *t* test. To test whether the home-practice outcome correlated directly with changes in visuomotor performance, we correlated individual changes between sessions in each measure reported below with the home-practice outcome. *r* and *p* values are indicated in the table.

### Baseline MRI measures and their relationship with practice-related visuomotor improvements

#### fMRI

**Figure 3.**
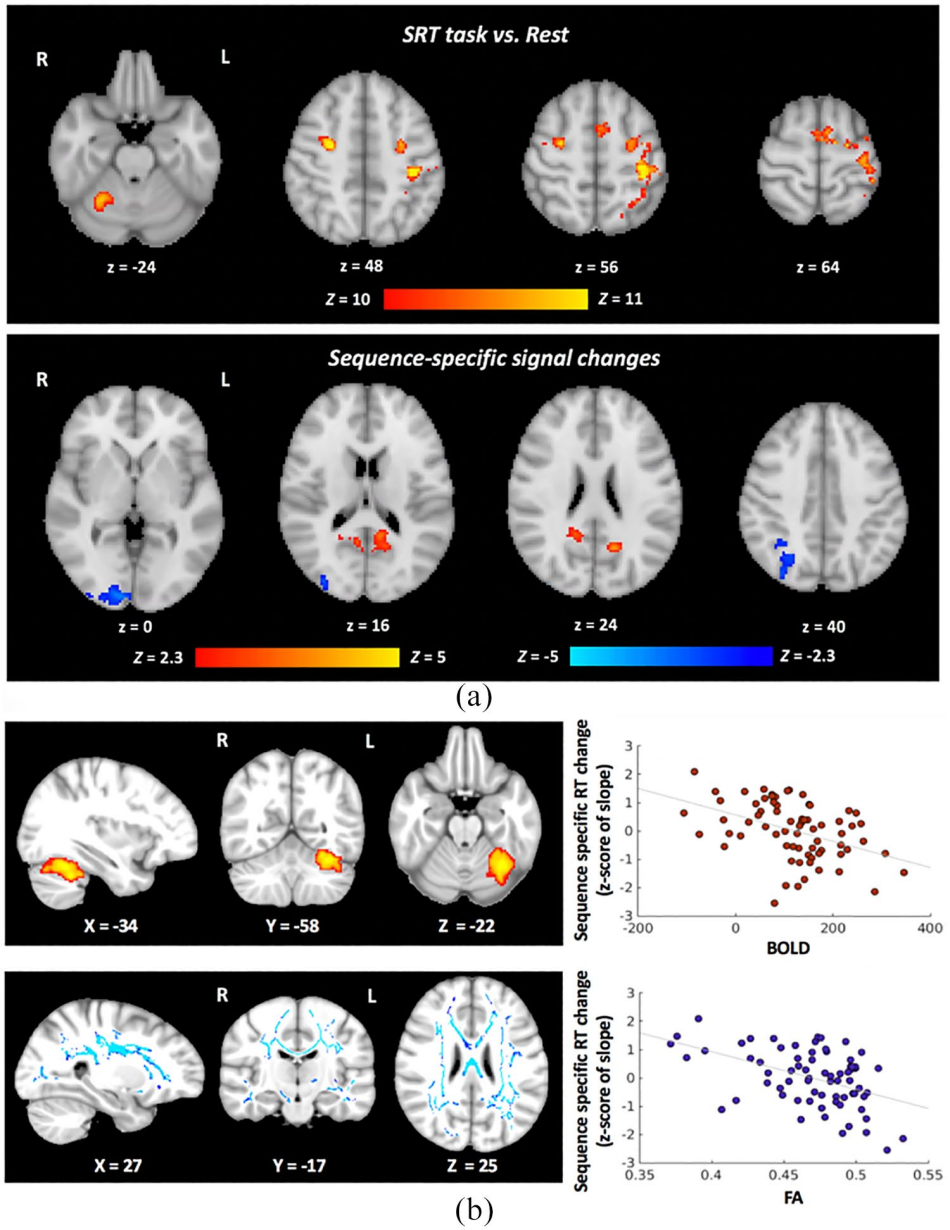
Group-level results of the SRT task-related functional MRI and functional and structural brain regions relevant for visuomotor performance improvements with home practice. (a) For the contrast SRT Task versus Rest, we identified task-relevant clusters in sensory motor and premotor cortices, in the right cerebellum and the visual cortex. Six clusters were retained as task-relevant regions of interest for the modelling approaches B and C: (1) left lateral pre- and postcentral gyrus (peak coordinates in the MNI space: –38, –32, 54; cluster size: 887 voxels); (2) left medial precentral gyrus (peak coordinates: –10, 2, 63; cluster size: 236 voxels); (3) right lateral precentral gyrus (peak coordinates: 28, –6, 46; cluster size: 195 voxels); (4) right cerebellar lobule VI (peak coordinates: 28, –52, –26; cluster size: 195 voxels); (5) right cerebellar lobule VIII (peak coordinates: 14, –62, –48; cluster size: 42 voxels); and (6) right visual cortex V5 (peak coordinates: 44, –68, 4; cluster size: 40 voxels). For the Sequence-specific contrast, signal changes were observed in three clusters: (1) activation increases in the right precuneus (peak coordinates: 8, –54, 14; cluster size: 541 voxels); (2) activation decreases in the right visual cortex V1 (peak coordinates: 10, –94, –2; cluster size: 627 voxels); and (3) activation decreases in the right intra-parietal sulcus (peak coordinates: 32, –52, 36; cluster size: 366 voxels). These three clusters were retained as task-relevant regions of interest for the modelling analysis approaches B and C. In blue we indicate regions with a reduction of the BOLD signal; in red colour, we indicate regions with an increase in the BOLD signal. (b) Localised correlations between the home-practice outcome and the baseline functional activity (the BOLD signal change in contrast Task vs Rest, *top*) and the baseline white matter microstructure (FA, *bottom*). Correlations were localised with group-level analyses using the training set (*n* = 71) only. Scatter plots show the relationship between the MRI measures in those regions and home-practice outcomes (*z*-standardised) in the training set. SRT: serial reaction time; BOLD: blood oxygenation level dependent; FA: fractional anisotropy; RT: reaction time; R: right; L: left.

In the training set, we identified SRT task–relevant regions, that is, bilateral motor, premotor, visual and somatosensory cortices, as well as the right cerebellum (Figure 3(a), top). Sequence-specific signal changes were observed in the posterior parietal and visual cortices (Figure 3(a), bottom).

When testing the relationship between functional responses in the SRT Task > Rest contrast and Sequence-specific RT changes, that is, the home training outcome, we found a significant correlation in the left cerebellar lobule VI (peak voxel coordinates in the MNI space: –36, –60, –24; cluster size: 722 voxels), where the BOLD signal change at baseline correlated with faster performance improvements (Figure 3(b), top). We did not find regions that showed the opposite relationship.

#### Structural MRI and resting perfusion MRI

In the training set, there was no significant correlation between baseline MRI measures of grey matter (GM) integrity (GM volume and magnetisation transfer ratio (MTR)) or CBF and Sequence-specific RT changes. However, higher fractional anisotropy (FA) values in a wide range of white matter (WM) tracts, including the corticospinal tract, corpus callosum, and longitudinal fasciculi, correlated significantly with the home-practice outcome, that is, higher FA values were associated with faster improvements in RT with practice (Figure 3(b), bottom).

### Predictors of performance improvements with home practice

#### Variable selection and parameter estimation (training set)

**Figure 4.**
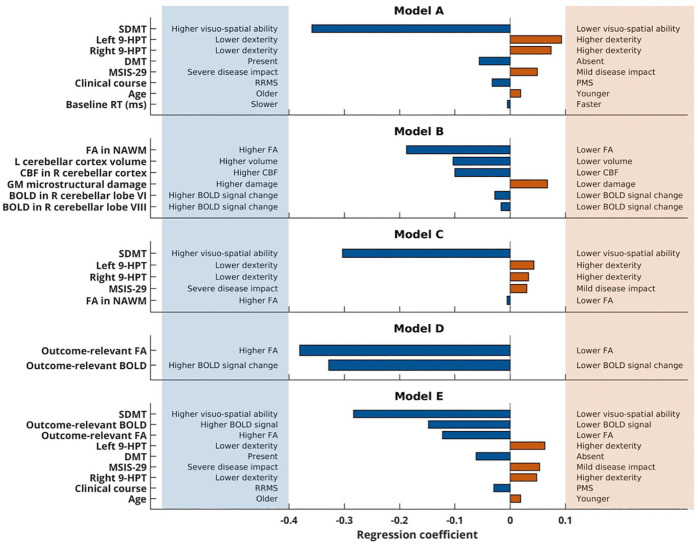
Predictive models. Predictors that were retained in the models and their respective contribution (regression coefficient) are shown on the *y*- and on the *x*-axis, respectively. The signs of the regression coefficient depend on the scaling of the predictor variable and the home-practice outcome (the negative slope reflects faster decreases in the reaction time). The lowest model error (MSE) in the validation set was found in model A, which was then evaluated in the test set. SDMT: Symbol Digit Modalities Test; 9-HPT: nine-hole peg test; DMT: disease-modifying treatment; MSIS-29: 29-item Multiple Sclerosis Impact Scale; RRMS: Relapsing-remitting MS; PMS: progressive (both primary and secondary) multiple sclerosis; RT: reaction time; FA: fractional anisotropy; NAWM: normal-appearing white matter; CBF: cerebral blood flow; GM: grey matter; BOLD: blood oxygenation level dependent (signal change).

Tables 1 and 2 list the clinical and MRI variables that were considered in the five modelling approaches for the predictive analysis. In each resulting model, between 3 and 13 variables were retained and contributed to the model to varying extents (Figure 4).

#### Model selection (validation set)

For each model, we calculated the model error (MSE). The lowest model error in the validation set was found in model A (model A: 1.16, model B: 1.33, model C: 1.28, model D: 1.63, and model E: 1.23).

#### Model evaluation (test set)

**Figure 5.**
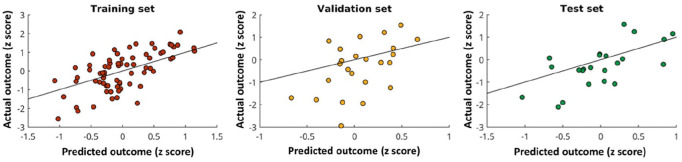
Model evaluation. Scatter plots showing the relationship between the predicted (from the model) home-practice outcome and the actual outcome in the training (*left*), validation (*middle*) and test (*right*) sets. The black line is the line of equality and indicates where the data points would lie in the case when the MSE is equal to 0.

Model A was applied to the independent test set. The home-practice outcome predicted by model A significantly correlated with the actual outcome (*r*(21) = 0.66, *p* < 0.0001; Figure 5) and could explain 44% of the variance.

Based on these model parameters (regression coefficients in Figure 4), the variables in model A predicted individual improvements with practice in upper limb performance according to the relationship

predictedoutcome(zscore)Y=Xβ

where *X* is a row vector of predictor values and β is a column vector of their respective beta coefficients (Table 4).

**Table 4.** Calculation of the predicted outcome with model A.

β_1_ = –0.360	*X*_1_ = ((score in SDMT – 52.9)/10.7)
β_2_ = +0.093	*X*_2_ = ((log[time in left 9-HPT (s)) – 3.22)/0.321]
β_3_ = +0.074	*X*_3_ = ((log[time in right 9-HPT (s)) – 3.20)/0.316]
β_4_ = –0.056	*X*_4_ = ((DMT^a^ – 0.282)/0.453)
β_5_ = +0.049	*X*_5_ = ((score in MSIS-29 – 66.2)/31.4)
β_6_ = –0.032	*X*_6_ = ((disease course^b^ – 0.803)/0.401)
β_7_ = +0.019	*X*_7_ = ((age – 44.8)/9.93)
β_8_ = –0.005	*X*_8_ = ((RT in SRT task (ms) – 383)/57.5)

SDMT: Symbol Digit Modalities Test; 9-HPT: nine-hole peg test; DMT: disease-modifying treatment; MSIS-29: 29-item Multiple Sclerosis Impact Scale; RT: reaction time; SRT: serial reaction time; RRMS: relapsing-remitting multiple sclerosis; MS: multiple sclerosis.

The regression coefficient for each predictor is provided, along with the required equation for calculating each predictor via *z* standardisation.

a 1 if on DMT and 0 if not on DMT.

b 1 if RRMS and 0 if progressive MS.

## Discussion

By combining baseline demographic, clinical and neuroimaging data, this study identified predictors of visuomotor performance improvements with training in individual MS patients. The strongest predictors were age, clinical characteristics such as disease duration, visuo-spatial abilities, upper limb dexterity and self-evaluated disease impact, and the presence of disease-modifying treatment (DMT). MRI metrics did not predict the training outcome over and above these measures, but variation in brain activity and WM microstructure in regions relevant to the practised task explained individual differences in the training outcome.

### Visuomotor training to probe upper limb functional recovery

A 4-week standardised visuomotor training intervention probed brain plasticity underlying functional recovery. Compliance was high, with a dropout rate of less than 20%. Patients varied in the number of trained days, but this did not affect the outcome measure.

Interventional studies rely on the patient’s compliance with the intervention procedures. We monitored compliance by recording the log file of each session of intervention and by asking the patients to complete a diary to record specific deviations from the daily protocol or factors affecting the practice. We mitigated the effect of variability in task execution by defining an outcome measure that is independent of the total number of practice sessions completed.

While, as a group, patients significantly improved performance with practice, the training outcome varied considerably between patients, reflecting the heterogeneity in functional recovery and rehabilitation outcomes observed in the real-life setting. The distribution of performance improvements (Figure 5) suggested a *continuum* rather than distinct groups of responders *versus* non-responders. Therefore, our modelling predicted the extent of performance improvement, without attempting an arbitrary classification of patients into groups.

Our contrast-derived training outcome, that is, slope of Sequence-specific RT changes, allowed us to limit the confounding effect of attention, fatigue or motivation on performance, thus capturing more stable changes in visuomotor performance.^21^ Although our intervention was not individually tailored, as it would be in clinical rehabilitation, it facilitated the generalisability and interpretability of the results by allowing all patients to experience the same, controlled training conditions. Indeed, the biological processes underlying clinically induced or experimentally driven improvements in performance largely overlap. First, rehabilitation leads to functional recovery that, in most cases, is sub-served by brain changes similar to those occurring with skill learning.^9^ Second, Sequence-specific improvements with training, closely reflecting task-oriented practice in rehabilitation, rely on systems involved in visuomotor integration and learning of movement sequences, functions that are relevant in rehabilitation.^3^ Although this study was not designed to test explicitly a generalisation of the effect of our intervention on routinely used clinical measures, changes in hand dexterity that accompanied performance improvements with practice suggest a possible, although small, clinical benefit of our experimental intervention.

### Predictors of performance improvements

We used a statistical learning approach to identify predictors of visuomotor performance improvements, as a conventional linear regression could lead to overfitting, that is, the resulting model could work well for the data that were used to establish the model, but not for independent data.

Within the most successful predictive model (model A), the strongest predictor was the performance of Symbol Digit Modalities Test (SDMT), a measure of visuo-spatial skills and speed of processing that is considered to be a powerful tool to assess cognition in MS.^22^ Patients with higher SDMT performance showed faster visuomotor performance improvements with practice, extending the relevance of cognitive reserve from normal motor learning^23^ to functional recovery.

Higher levels of hand dexterity predicted performance improvements, with scores of both hands contributing independently to the model. Although right-hand dexterity is directly relevant to the execution of the study intervention, left-hand dexterity in MS may also reflect the function of the left motor regions, thus contributing to explain the independent predictive role of the left upper limb function in performance improvements of the right upper limb.^24^

Our results also suggest that younger age, higher pre-morbid reserve, lower disease burden, as assessed formally and by self-report, and modulation of inflammation predict patients who will improve performance with practice. Since our intervention relies on brain plasticity, these factors can also act as determinants of the intervention success, as they can be associated with building,^25^ maintaining^26^ and exploiting^27^ plastic reserve in the patients. By contrast, pathological MS changes, from brain atrophy to vascular abnormalities, can contribute to reduce plastic reserve in patients with higher disease burden, adding to the effect of age on brain health.^5,28^

### MRI correlates of performance improvements

Performance improvements with training correlated significantly with baseline group-level structural and fMRI measures. Higher FA in the longitudinal fasciculi, corticospinal tracts and corpus callosum was significantly associated with better home training outcome. These regions are important for visuomotor integration, inter-hemispheric communication and motor execution, functions all relevant to our visuomotor task.^29^ These effects were widespread across white matter, suggesting that, along with the integrity of specific regions, the overall microstructural health, that provides the substrate for local and long-range connectivity and that is disrupted by MS damage,^30,31^ is beneficial to support full recovery.

Patients with stronger cerebellar activation, as measured by BOLD fMRI during the SRT task, showed greater improvements with practice. A localised brain–behaviour relationship was found in cerebellar lobule VI, which is functionally connected with the contralateral higher motor control regions.^32^ While the relationship between training outcomes and baseline SRT–related signal changes in the cerebellum may result from a simple modification of performance,^33^ it could also suggest that stronger or more intact error processing function leads to better training outcomes.^34^

At least within the range of damage and length of training studied here, brain MRI data did not predict the outcome over and above demographic and clinical data, confirming the difficulty in translating directly results from MRI group-level analyses to the individual patient and clinical practice, and highlighting the importance of developing novel biologically informative MRI-based metrics to increase the potential of neuroimaging for single-subject prediction.^35^ Some aspects of damage in our population, for example, those revealed by other MRI methods (e.g., MR spectroscopy) or within other anatomical sites (e.g., the spinal cord and optic nerve), may not have been fully captured. However, we aimed for a comprehensive, yet feasible baseline characterisation of the brain function and structure in a single MRI session, and selected MRI methods and measurements, whose biological informativeness for MS damage, repair and systems-level plasticity is well established.^3^ While we aimed to predict performance improvements from a single baseline assessment, it is possible that the analysis of longitudinal clinical and MRI data could identify MRI predictors of functional recovery.

## Conclusion

A comprehensive formal, as well as self-reported, baseline clinical assessment offers a reliable indication of the likely extent of recovery of the visuomotor function in individual MS patients, at least within the range of disabilities, times and activities studied here. Residual abilities are retained functions, sub-served by the individual functional reserve, that is, the remaining capacity of the brain to cope with an increased behavioural demand.^36^ Our results highlight the importance of extending the routine clinical assessment of current disability to include measures of residual abilities relevant for recovery. Prediction of functional recovery in individual patients can provide valuable information at an early stage regarding the likelihood of response to standard rehabilitation interventions, as well as the stratification of patients for recovery-oriented clinical trials. The identification of structural and functional imaging correlates of performance improvements in a large cohort of patients provides a strong rationale for further, more targeted exploration of neuroimaging predictors of recovery.

## Supplemental Material

MSJ943788_supplemental_figure – Supplemental material for Predictors of training-related improvement in visuomotor performance in patients with multiple sclerosis: A behavioural and MRI study Supplemental material, MSJ943788_supplemental_figure for Predictors of training-related improvement in visuomotor performance in patients with multiple sclerosis: A behavioural and MRI study by Ilona Lipp, Catherine Foster, Rachael Stickland, Eleonora Sgarlata, Emma C Tallantyre, Alison E Davidson, Neil P Robertson, Derek K Jones, Richard G Wise and Valentina Tomassini in Multiple Sclerosis Journal

MSJ943788_supplemental_material – Supplemental material for Predictors of training-related improvement in visuomotor performance in patients with multiple sclerosis: A behavioural and MRI study Supplemental material, MSJ943788_supplemental_material for Predictors of training-related improvement in visuomotor performance in patients with multiple sclerosis: A behavioural and MRI study by Ilona Lipp, Catherine Foster, Rachael Stickland, Eleonora Sgarlata, Emma C Tallantyre, Alison E Davidson, Neil P Robertson, Derek K Jones, Richard G Wise and Valentina Tomassini in Multiple Sclerosis Journal
