# Supplementary material for: Predictors of training-related improvement in visuomotor performance in patients with multiple sclerosis: A behavioural and MRI study
Source: Mult Scler. 2020 Aug 4;27(7):1088–101. doi: 10.1177/1352458520943788 (PMC8151554; doi:10.1177/1352458520943788)
Supplement: MSJ943788_supplemental_figure – Supplemental material for Predictors of training-related improvement in visuomotor performance in patients with multiple sclerosis: A behavioural and MRI study [file MSJ943788_supplemental_figure.pdf]

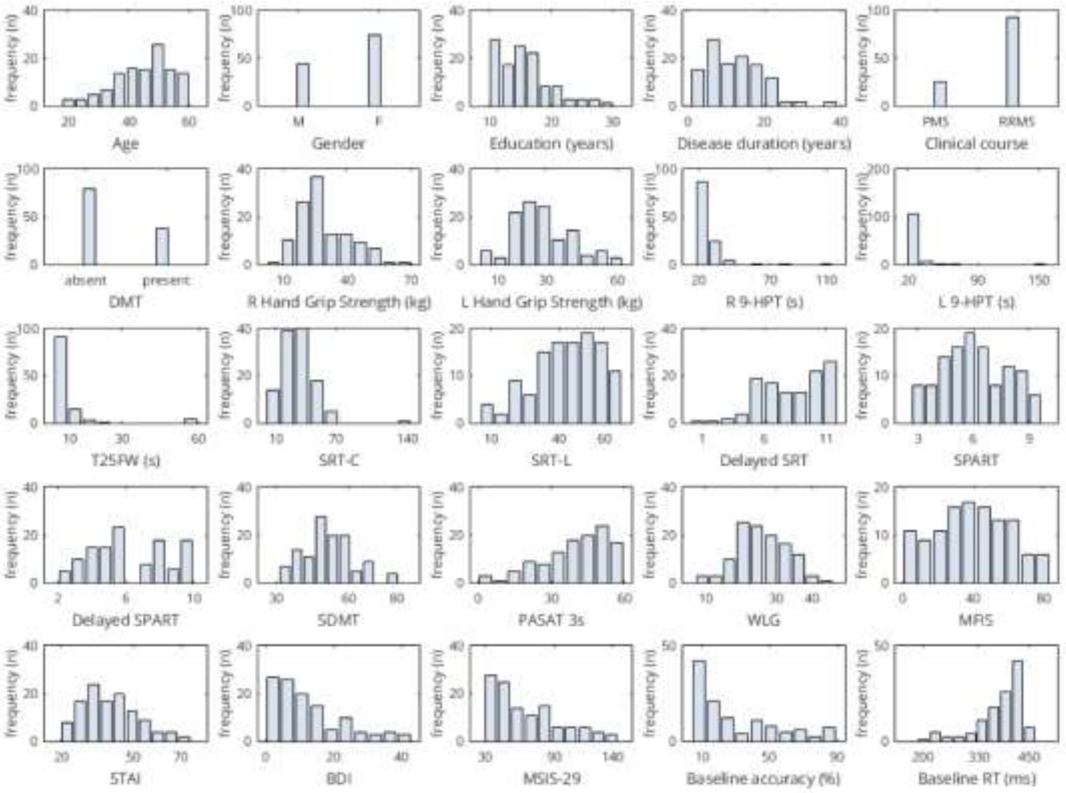

Supplementary Material – Figure legend

Figure S1. Frequency distributions of demographic and clinical characteristics. For each measure, a histogram is provided across the entire sample (n = 118). The Y axis indicates frequency; the X axis indicates the range or groups of measures relevant for the graph. ***Abbreviations:*** RRMS = relapsing-remitting MS, PMS = progressive MS, DMT = disease-modifying treatment, R = right, L = left, 9-HPT = 9-Hole Peg Test, T25-FW = timed 25 foot walk, SRT-C = Selective Reminding Test Consistent Retrieval, SRT-L = Selective Reminding Test Long-term Storage, SPART = Spatial Recall Test, SDMT = Symbol Digit Modalities Test, PASAT = Paced Auditory Serial Addition Test, WLG = Word List Generation, MFIS = Modified Fatigue Impact Scale, STAI = State Trait Anxiety Inventory, BDI = Beck Depression Inventory, MSIS-29 = Multiple Sclerosis Impact Scale, RT = reaction time.
