# Supplementary material for: Predictors of training-related improvement in visuomotor performance in patients with multiple sclerosis: A behavioural and MRI study
Source: Mult Scler. 2020 Aug 4;27(7):1088–101. doi: 10.1177/1352458520943788 (PMC8151554; doi:10.1177/1352458520943788)
Supplement: MSJ943788_supplemental_material – Supplemental material for Predictors of training-related improvement in visuomotor performance in patients with multiple sclerosis: A behavioural and MRI study [file MSJ943788_supplemental_material.pdf]

Supplementary Material – Manuscript

Visuomotor task

During the serial reaction time (SRT) task, participants were presented with visual stimuli on a computer screen and asked to respond to their location as quickly and accurately as possible by pressing the corresponding key on a keypad with one of the four fingers on the right hand (index to little finger) (**Fig. 1A**).

For the baseline SRT task, each of the Sequence blocks consisted of four repeats of eight stimuli and lasted for 16 seconds. Random blocks (once every third block) were introduced to account for non-specific variability in task performance with practice[1]; each block lasted for 16 seconds. Rest blocks lasted on average for 15 seconds and presented a static image that matched the task, in order to control for visual input.

For the home training, the Sequence condition included 14 blocks, each consisting of three repeats of 16 stimuli. The sequence was matched in difficulty to the randomly generated sequences[2, 3]. After every two Sequence blocks, there was a Random block, where 48 stimuli appeared in a random order, not allowing the same stimulus to appear twice consecutively. In both conditions, stimulus duration was 325 ms with an inter-stimulus interval of 175 ms. Each block lasted for 24 seconds and was followed by a Rest period of 10 seconds. One session of practice lasted for approximately 15 minutes.

At baseline and for the home training, we allowed a time window for anticipation of up to 500 ms before stimulus onset.

MRI acquisition and analysis

Macrostructural damage

White matter (WM). WM lesions were identified on T2-weighted images [proton-density (PD)/T2-weighted dual echo sequence, TEs = 9.0/80.6 ms, TR = 3000 ms, flip angle = 90°,

resolution = 0.94x0.94x4.5 mm, 36 slices, 3 mm thickness, 1.5 mm gap], also consulting the PD-weighted images and the T2-weighted fluid-attenuated inversion recovery (FLAIR) sequence, acquired in the same orientation (TE = 122 ms, TR = 9502 ms, inversion time = 2250 ms, flip angle = 90°, resolution = 0.86x0.86x4.5 mm). Lesions were semi-automatically segmented using Jim (v.6, Xinapse) on T2-weighted images.

Grey matter (GM). We acquired 3D Fast Spoiled Gradient-Recalled-Echo T1-weighted images (TE = 3ms, TR = 7.5ms, flip angle = 20°, matrix = 256x256x172, resolution = 1 mm^3) for GM volumetric analysis. After lesion filling[4], we obtained whole brain volume fraction, cortical and subcortical volume fractions (all in relation to intracranial volume), and average cortical thickness with Freesurfer's (v.5.3.0) *recon-all* pipeline. To localise correlations between home-practice outcome and GM volume, we conducted a voxel-based-morphometry analysis using SPM12 (http://www.fil.ion.ucl.ac.uk/spm/) in the training set. Group-level statistics were thresholded with a two-sided, cluster-level, family wise-error corrected threshold of $p < 0.05$.

Microstructural damage

GM. Magnetization transfer imaging (MTI) was acquired (Enhanced Fast Gradient Echo 3D, resolution = 0.94x0.94x1.9mm, TE = 1.8ms, TR = 26.7ms, flip angle = 5°, off-resonance pulse: 450° and 2khz off resonance). Firstly, we calculated voxel-wise magnetisation transfer ratio (MTR) with:

$$MTR = [(S_0-S_{MT})/S_0] \times 100$$

S_0 representing the signal without and S_{MT} representing the signal with the off-resonance pulse. The MTR image was brain extracted using BET from FSL (https://fsl.fmrib.ox.ac.uk/fsl/fslwiki) and non-linearly registered to the brain-extracted T1-weighted image using Elastix[5]. Then, the images were normalised to the MNI152 2 mm

template, using a warp determined through the T1-weighted image with ANTs Syn[6, 7]. The same procedure was applied to data from 27 healthy volunteers (38.1±11.0 years, 15 women). For each patient and each voxel, the MTR was classified as microstructurally damaged, if it lay 1.65 standard deviations below the mean of the healthy control sample (corresponding to a 1-sided threshold for $p < 0.05$). For each patient, we calculated the proportion of microstructurally damaged voxels in GM, which was defined using FAST from FSL (https://fsl.fmrib.ox.ac.uk/fsl/fslwiki) on the lesion filled T1-weighted image, thresholded at 80%. We used this proportion as a global measure of GM microstructural damage.

To localise correlations between home-practice outcome and GM microstructural damage, FSL randomise (https://fsl.fmrib.ox.ac.uk/fsl/fslwiki) was used on the MTR images in MNI space (5000 permutations, threshold-free-cluster-enhancement corrected at $p < 0.05$, two-sided).

WM. We acquired diffusion-weighted imaging (DWI) with 40 uniformly distributed directions (b value = 1200) taken from Camino (http://camino.cs.ucl.ac.uk/) and six non-diffusion weighted images at the beginning (resolution: 1.8x1.8x2.4mm, 57 slices, TE = 94.5ms, TR = 16000ms, flip angle = 90°). Data were processed using ExploreDTI (http://www.exploredti.com), including correction for head motion and distortions, reorientation of the diffusion encoding vectors[8] and modulation of the signal intensity by the determinant of the Jacobian of the transformation[9]. RESTORE[10] was used to estimate a robust diffusion tensor. Maps of fractional anisotropy (FA) and radial diffusivity (RD) were derived and normalised to MNI space. To localise correlations between home-practice outcome and WM microstructural damage, TBSS from FSL (https://fsl.fmrib.ox.ac.uk/fsl/fslwiki) was used. FA images were registered to the FMRIB58 FA template. A subject-specific skeleton was derived and thresholded at FA of 0.3. Using home-practice outcome as a regressor, permutation

tests were computed in the training set (5000 permutations, masked with the skeleton mask, $p < 0.05$ two-sided, corrected using threshold free cluster enhancement correction).

Normalisation of GM and WM microstructural measures. Voxel-wise z-scores using mean and standard deviation of values from healthy volunteers were calculated for the three microstructural metrics (MTR, FA, RD) of patients to obtain normalised measures of microstructural damage. These normalised measures were averaged (median) across comparable white matter locations within each patient's WM lesions and within normal appearing WM (NAWM), as defined by FAST segmented (80% thresholded) WM at least 5 mm away from lesions.

fMRI

SRT task related signal changes. Blood Oxygenation Level Dependant (BOLD)-weighted fMRI images (resolution = 3.4x3.4x3mm, TR = 3000ms, TE = 35ms, FOV/slice = 220mm, flip angle = 90°, 46 slices of 2 mm, 1 mm gap, 142 volumes) were acquired during SRT task. Pre-processing and first level analyses were carried out using FEAT from FSL (https://fsl.fmrib.ox.ac.uk). Four regressors were defined: Sequence, Random and linear change over time of Sequence and Random blocks. The two main contrasts of interest were: SRT task>Rest and Sequence-specific signal changes, reflecting activation changes over time that were stronger in the Sequence than in the Random condition.

To localise SRT task-relevant regions, group-level analyses were performed in the training set [voxel threshold of $z = 2.3$ ($z = 10$ for the SRT task > Rest contrast) two-sided, cluster threshold $p < 0.05$]. Significant clusters were retained as regions of interest (ROIs) for the statistical modelling and average SRT task-related BOLD signal changes within these ROIs entered the predictive modelling as predictors. The same group-level analysis approach was used to test

brain-behaviour correlations, using the individual home-practice outcome as a regressor. FSL Harvard-Oxford and Juelich histological atlases (https://fsl.fmrib.ox.ac.uk/fsl/fslwiki) were used to report anatomical locations.

Perfusion

CBF. We quantified CBF as an indicator of vascular health using multi-inversion time pulsed arterial spin labelling (ASL). We employed a PICORE QUIPSS II sequence with a dual-echo gradient-echo readout and spiral k-space acquisition (resolution = 3x3x8 mm, 22 slices, 7 mm thickness, 1 mm gap)[11]. We acquired 16 tag-control pairs each for short inversion times (400, 500, 600, 700 ms) and 8 tag-control pairs for long inversion times (1100, 1400, 1700 and 2000 ms) with QUIPSS II cut-off at 700 ms. A calibration (M_0) image was acquired to obtain the equilibrium magnetization of cerebrospinal fluid, needed for the quantification of CBF. A minimal contrast image was acquired with TE = 11ms, TR = 2000ms to correct for coil inhomogeneities. CBF was estimated using *oxford_asl* with partial volume correction[12] through the application of FAST-derived estimated GM probabilities. CBF maps were registered to the T1 structural scan following 6 DOF affine registration of the M_0 scan. Median CBF measures were extracted from Freesurfer-segmented regions (bilateral cerebral cortex, cerebellar cortex, caudate, putamen, thalamus, pallidus) and used as ROIs in the prediction analysis. To localise correlations with home-practice outcome FSL randomise[13] was used for group-level statistics (5000 permutations, threshold-free-cluster-enhancement corrected, $p<0.05$ two-sided).

References

1. Robertson EM. The serial reaction time task: implicit motor skill learning? *J Neurosci*. 2007; 27: 10073-5.

2. Curran T. Higher-order associative learning in amnesia: evidence from the serial reaction time task. *J Cogn Neurosci*. 1997; 9: 522-33.

3. Vaquero JM, Jimenez L and Lupianez J. The problem of reversals in assessing implicit sequence learning with serial reaction time tasks. *Exp Brain Res*. 2006; 175: 97-109.

4. Gelineau-Morel R, Tomassini V, Jenkinson M, Johansen-Berg H, Matthews PM and Palace J. The effect of hypointense white matter lesions on automated gray matter segmentation in multiple sclerosis. *Hum Brain Mapp*. 2012; 33: 2802-14.

5. Klein S, Staring M, Murphy K, Viergever MA and Pluim JP. elastix: a toolbox for intensity-based medical image registration. *IEEE Trans Med Imaging*. 2010; 29: 196-205.

6. Avants BB, Epstein CL, Grossman M and Gee JC. Symmetric diffeomorphic image registration with cross-correlation: evaluating automated labeling of elderly and neurodegenerative brain. *Med Image Anal*. 2008; 12: 26-41.

7. Ou Y, Akbari H, Bilello M, Da X and Davatzikos C. Comparative evaluation of registration algorithms in different brain databases with varying difficulty: results and insights. *IEEE Trans Med Imaging*. 2014; 33: 2039-65.

8. Leemans A and Jones DK. The B-matrix must be rotated when correcting for subject motion in DTI data. *Magn Reson Med*. 2009; 61: 1336-49.

9. Jones DK and Cercignani M. Twenty-five pitfalls in the analysis of diffusion MRI data. *NMR Biomed*. 2010; 23: 803-20.

10. Chang LC, Jones DK and Pierpaoli C. RESTORE: robust estimation of tensors by outlier rejection. *Magn Reson Med*. 2005; 53: 1088-95.

11. Warnert EA, Murphy K, Hall JE and Wise RG. Noninvasive assessment of arterial compliance of human cerebral arteries with short inversion time arterial spin labeling. *J Cereb Blood Flow Metab*. 2015; 35: 461-8.

12. Chappell MA, Groves AR, MacIntosh BJ, Donahue MJ, Jezzard P and Woolrich MW. Partial volume correction of multiple inversion time arterial spin labeling MRI data. *Magn Reson Med*. 2011; 65: 1173-83.

13. Winkler AM, Ridgway GR, Webster MA, Smith SM and Nichols TE. Permutation inference for the general linear model. *Neuroimage*. 2014; 92: 381-97.
